# Conference Calendar

**DOI:** 10.1002/cfg.403

**Published:** 2004-04


**Comparative and Functional Genomics**
*Comp Funct Genom* 2004; **5**: 292–294.
Published online in Wiley InterScience (www.interscience.wiley.com). **DOI:** 10.1002/cfg.403

### Conference Calendar

September–November 2004

### Animal models

CSHL — Mouse Molecular Genetics, 1–5 September

**http://meetings.cshl.org/2004/2004mouse.htm**

ISAG — 29th International Conference on Animal Genetics, 11–16 September

**http://www2.kobe-u.ac.jp/%7Eisag2004/**

Chicken Genome Sequence: Impact and Applications Symposium, 13–14 November

**http://www.avigenics.com/symposium1.htm**

### Bacteria

SGM — 155th Meeting of the Society for General Microbiology, 6–9 September

**http://www.sgm.ac.uk/meetings/MTG PAGES/Tcd.cfm**

ASM — 5th International Conference on Extremophiles, 19–23 September

**http://www.asm.org/Meetings/index.asp?bid=19177**

ESF IAFG — Environmental Genomics and Environmental Metagenomics, 18–20 November

**http://www.eez.csic.es/genomics/**

### Bioinformatics

15th European Conference on Machine Learning/8th European Conference on Principles and Practice of Knowledge Discovery in Databases, 20–24 September

**http://ecmlpkdd.isti.cnr.it/index.shtml**

CSHL/WT — Genome Informatics, 22–26 September

**http://meetings.cshl.org/2004/2004infouk.htm**

IBC — BioDigital 2004, 13–15 October

**http://www.biodigital.de/**

NAS — Arthur M. Sackler Colloquium: Frontiers in Bioinformatics: Unsolved Problems and Challenges, 15–17 October

**http://www4.nationalacademies.org/nas/nashome.nsf/urllinks/NAS-58MTTC?OpenDocument**

TIGR — 7th Annual Conference on Computational Genomics, 21–24 October

**http://www.tigr.org/conf/cg/**

WSEAS — 5th International Conference on Mathematics and Computers in Biology and Chemistry, 15–17 November

**http://www.worldses.org/conferences/2004/iran/mcbc/index.html**

### Evolution/comparative genomics

1st Baikal Workshop on Evolutionary Biology, 6–11 September

**http://lin.irk.ru/1BWEB/**

8th Evolutionary Biology Meeting at Marseille, 22–24 September

**http://www.up.univ-mrs.fr/evol/congres/**

### Functional genomics

ASM — Functional Genomics and Bioinformatics Approaches to Infectious Disease Research, 6–9 October

**http://www.asm.org/Meetings/index.asp?bid=22657**

Copyright © 2004 John Wiley & Sons, Ltd.

EMBO/EMBL — Functional Genomics: Exploring the Edges of Omics, 16–19 October

**http://www.embl.de/conferences/Omics04/**

### General

15th International Chromosome Conference, 5–10 September

**http://www.the-conference.com/2004/iccxv/index.php**

ASHG — Annual Meeting, 26–30 October

**http://genetics.faseb.org/genetics/ashg/menu-annmeet.shtml**

### Pharmacogenomics

Society for Biomolecular Screening Annual Conference, 11–15 September

**http://www.sbsonline.org/**

Evolution Summit 2004, 20–22 September

**http://www.evolution-summit.com/**

3rd Annual Meeting of the International Society of Pharmacogenomics (ISP), 30 September–4 October

**http://biol.prospective-conf.u-nancy.fr/Welcome.htm**

CHI — Genomics on Target, 8–12 November

**http://www.genomicsontarget.com**

CSHL/WT — Pharmacogenomics, 18–21 November

**http://meetings.cshl.org/2004/2004pharm.htm**

### Plants

Plant GEMS 3, 22–25 September

**http://plant-gems.org/plant_gems/index.html**

CSHL — Plant Genomes: From Sequence to Phenome, 9–12 December

**http://meetings.cshl.org/2004/2004plants.htm**

### Proteomics

CHI — Protein Biomarkers, 30 August–1 September

**http://www.healthtech.com/conference-fall04.asp**

3rd International and 28th European Peptide Symposium, 5–10 September

**http://kenes.com/28eps/index.html**

### Structural genomics

IAPSAP — 15th Meeting on Methods in Protein Structure Analysis, 29 August–2 September

**http://depts.washington.edu/biowww/mpsa2004/**

EMBO — Conference on Structures in Biology, 10–13 November

**http://www.embl-heidelberg.de/conferences/StructBiol04/**

3rd International Conference on Structural Genomics, 17–21 November

**http://www-nmr.cabm.rutgers.edu/icsg2004/**

### Systems biology

BTK — Developing concepts for systems biology, 3–6 September

**http://mudshark.brookes.ac.uk/BTK/**

5th International Conference on Systems Biology, 9–13 October

**http://www.icsb2004.org/**

### Transcriptomics

CSHL — Dynamic Organization of Nuclear Function, 29 September–3 October

**http://meetings.cshl.org/2004/2004nucleus.htm**

Copyright © 2004 John Wiley & Sons, Ltd.

### Biotech industry

BIOPHEX 2004: Post-discovery Through Commercialization, 28–30 September

**http://www.biophex.com/**

Biotechnology 2004, 17–22 October

**http://www.conicyt.cl/IBS2004/**

### Key

ASHG — American Society for Human Genetics: **http://www.ashg.org**
ASM — American Society for Microbiology: **http://www.asm.org**
BTK — BioThermoKinetics Study Group: **http://dbk.ch.umist.ac.uk/btk/home.htm**
CHI — Cambridge Healthtech Institute: **http://www.healthtech.com**
CSHL — Cold Spring Harbor Laboratories: **http://meetings.cshl.org/index.htm**
EMBL — European Molecular Biology Laboratory: **http://www.embl-heidelberg.de/**
EMBO — European Molecular Biology Organisation: **http://www.embo.org/**
ESF IAFG — European Science Foundation, Integrated Approaches for Functional Genomics: **http://www.functionalgenomics.org.uk/**
IAPSAP — International Association for Protein Structure Analysis and Proteomics: **http://www.iapsap.bnl.gov/**
IBC — IBC Conferences: **http://www.ibc-lifesci.com**
ISAG — International Society for Animal Genetics: **http://www.isag.org.uk/**
NAS — National Academy of Sciences: **http://www4.nationalacademies.org/nas/nashome.nsf**
SGM — Society for General Microbiology: **http://www.sgm.ac.uk**
TIGR — The Institute for Genomic Research: **http://www.tigr.org**
WSEAS — World Scientific and Engineering Academy and Society: **http://www.worldses.org**
WT — Wellcome Trust: **http://www.wellcome.ac.uk/**

The Conference Calendar is a listing of forthcoming conferences of interest to our readership. We provide the title, dates and web address of each conference, to enable readers to find out more information. Inclusion does not imply endorsement by the journal. If you know of a relevant conference, please contact the Managing Editor.

Copyright © 2004 John Wiley & Sons, Ltd. 

